# Beyond Ubiquity: Scale-dependent patterns of tardigrade diversity on the Iztaccíhuatl volcano

**DOI:** 10.1371/journal.pone.0343098

**Published:** 2026-03-04

**Authors:** Alba Dueñas-Cedillo, Francisco Armendáriz-Toledano, Rodolfo J. Cancino-López, Jazmín García-Román, Enrico Alejandro Ruiz

**Affiliations:** 1 Departamento de Zoología, Laboratorio de Ecología, Escuela Nacional de Ciencias Biológicas, Instituto Politécnico Nacional, Ciudad de México, México; 2 Departamento de Zoología, Colección Nacional de Insectos, Instituto de Biología, Universidad Nacional Autónoma de México, Ciudad de México, México; 3 Facultad de Ciencias Forestales, Universidad Autónoma de Nuevo León, Linares, Nuevo León, México; University of Braga, PORTUGAL

## Abstract

The diversity of tardigrade communities has been related with variables, such as habitat type, litter type, elevation, among others. However, the integration of variables in a multiscale context has been little explored, so this study analyzed tardigrade diversity and community composition across multiple ecological scales—moss substrates (rock, soil, bark), landscapes, and elevation zones—in a montane ecosystem. Mosses on tree bark harbor the highest species richness, including several substrate-specific taxa, while mosses on soil hosts unique species not found elsewhere. Mosses on rocks share species with soil mosses but lacks exclusive taxa. Among landscapes, the coniferous forests (mixed, *Abies religiosa*, and *Pinus hartwegii*) exhibit high species richness and community similarity, with distinct local assemblages characterized by exclusive species within each forest type. Three generalist species were ubiquitous across all landscapes. Elevation analysis reveals maximal tardigrade richness and abundance in the alpine zone, with the nival zone supporting fewer species, mostly a subset of alpine taxa, and hosting a few unique species likely adapted to harsher conditions. Beta diversity analyses indicated that species turnover rather than nestedness predominantly drives community dissimilarities across substrates and habitats. These findings highlight the importance of considering scale-dependent patterns in understanding tardigrade distribution in complex montane environments.

## Introduction

The biological communities’ scale and pattern problem refers to how ecological patterns, such as species distribution, biodiversity, and interactions, vary across spatial and temporal scales [1]. This concept is crucial in ecology because patterns observed at one scale may not necessarily apply at another [2–4]. Microinvertebrates, including nematodes, rotifers, copepods, and tardigrades, exhibit distinct patterns shaped by both small-scale biotic interactions and large-scale environmental factors [5–7]. Fine-scale heterogeneity often governs local diversity and coexistence, while large-scale factors, such as geographic distance or climatic variation, influence species turnover and community composition across regions [8–12]. Multiscale analyses help disentangle the relative importance of these drivers, revealing patterns that would remain obscured in single-scale studies [6,7,13,14].

The multiscale approach is especially relevant in complex landscapes like elevation gradients, where rapid shifts in abiotic and biotic conditions can amplify scale-dependent effects. For microinvertebrates—organisms with high sensitivity to environmental changes and key functional roles in ecosystems—understanding these multiscale processes is essential for predicting their responses to environmental stressors and informing effective conservation strategies [6,7,15].

In microinvertebrates, altitude plays a key role in shaping species richness, with two main patterns observed: 1) decreasing richness at higher altitudes, and 2) greater richness at intermediate elevations [16,17]. While these patterns have been studied in larger organisms (>2 mm), mesofauna (<2 mm) remains less explored [5]. Studies on nematodes and rotifers show contrasting results, with nematode richness peaking at intermediate elevations [18], while rotifers show a decline at higher altitudes with species turnover [5].

Tardigrades, a small hydrophilic phylum, exhibit distinct elevation distribution patterns, with variations in species richness, abundance, and composition along elevation gradients [6,19–23]. A general elevation trend in tardigrade communities is a higher species richness at medium altitudes (*e.g.,* 1,500–3,000 m), following a hump-shaped pattern, which has been linked with optimal moisture conditions, high microhabitat diversity (mosses, lichens, soils) or reduced competition and predation [2,14,24,25]. The other pattern is a decreasing richness with increasing altitude, or species diversity declines at higher elevations, likely due to extreme temperature fluctuations, reduced vegetation cover, habitat availability, or increased UV exposure and desiccation risk [8,26]. Another trend is the presence of high-altitude specialists, observed in certain tardigrade species well-adapted to alpine and polar conditions, surviving in glaciers, permafrost, and high-altitude mosses [27–29].

Besides altitude, factors like vegetation, humidity, rainfall, and temperature influence tardigrade richness and habitat selection, with higher species richness found in dense, humid forests, and lower richness in open, xeric areas [11,30–32].

In a smaller scale, most terrestrial tardigrades are found many substrates, in mosses where moisture is a major driver of species distribution [33,34]. Moisture retention is influenced by moss morphology and habitat structure [35,36], with epiphytic mosses on tree bark generally supporting higher species richness than soil mosses due to better hydration [34,37]. Despite this, few studies have compared tardigrade diversity across moss substrates under similar conditions.

In Mexico, tardigrade research has primarily focused on faunistic and taxonomic aspects, with approximately 77 species recorded to date [38–42]. The Trans-Mexican Volcanic Belt (TMVB), one of the most diverse biogeographical regions [43], offers ideal conditions for ecological studies due to its elevation range and habitat variety. In the present study, over a wide elevation interval from 2700 to 4500 m asl, on the volcano Iztaccihuatl (located in TMVB), we assessed whether patterns of diversity of tardigrade assemblages in mosses are dependent on ecological scale. We estimated alpha and beta diversity and characterized the structure and composition of the assemblages at three ecological scales (habitat, landscape and region).

We sampled mosses from different substrate types (habitat scale)—moss growing on rocks, moss growing on tree bark, and moss growing on soil—, in different landscapes (landscape scale)—mixed forest (*Pinus*/*Hesperocyparis,*/*Quercus*), *Abies religiosa* forest, *Pinus hartwegii* forest, alpine grassland and tundra—, along two elevation zones (regional scale), the alpine zone (2,700−4,000m asl) and the nival zone > 4,000 m asl.

## Materials and methods

### Study site

**Fig 1:**
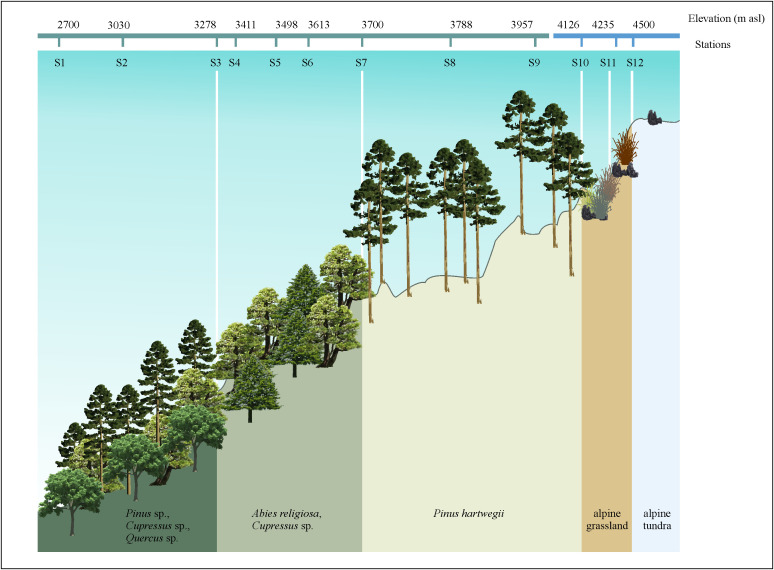
Study area located on the southwestern slope of Iztaccihuatl volcano in central Mexico. Landscape profile, showing collection stations and elevation for each station.

The study area was located on the southwestern slope of Iztaccihuatl volcano in the Sierra Nevada, TMVB (Fig 1). Within the study area, five landscapes, has been recognized [44-50], and were sampled (Fig 1).

1 Mixed Forest (2,500−2,800 m asl), dominated by *Pinus*, *Hesperocyparis*, and *Quercus* species [44]. 2 Mesophilic forest (2,800−3,500 m asl) dominated by *Abies religiosa*, accompanied by a few individuals of *Alnus jorullensis*, *Eupatorium pascuarense*, *Senecio angustifolius*, *Hesperocyparis* sp., and, to a lesser extent, *Quercus* spp. and *Pinus* spp. [44–46]. 3 *Pinus hartwegii* forest (3,700−4,000 m asl) dominated by *Pinus hartwegii*, along with shrubs like *Lupinus montanus*, *L. mexicanus*, and tufted grasses such as *Festuca tolucensis*. It represents the forest limit zone (timberline), marking the highest elevation reached by woody plants, and this is also a transition zone between coniferous forest and alpine landscape, and between alpine (2,700−4,000 m asl) and nival (>4,000 m asl) zones [44–49]. 4 Alpine Grassland (3,900−4,300 m asl) dominated by *Senecio procumbens*, *Calamagrostis tolucensis*, and *Packera bellidifolius*, along with mosses and lichens growing on rocks and soil [45,46,50]. 5. Alpine Tundra (>4,300 m asl) dominated by mosses and lichens on rock and soil, with no shrubs present [45,51,52]. These five landscapes correspond to two elevational zones, the alpine (S1–S9; 2,700−4,000 m asl) and the nival zones (S10–S12; 4,126−4,500 m asl) (Fig 1).

### Field sampling and processing

We defined 12 different sampling sites (S1–S12), distributed along the elevation gradient (2,700−4,500 m asl), which included the five landscapes described above (Table 1). At each site, we selected mosses of at least 300 cm^2^ of area growing in different substrates (rock, bark, and soil). Each moss was considered a sampling unit. To reduce the possibility of biased sampling, six replicates of 4 cm^2^ from each sampling unit were randomly collected. Along the 12 sites, 57 sampling units were considered, and 342 moss replicates were collected. Each moss sample was stored in a paper envelope and dried out for two weeks at room temperature. In the laboratory, moss samples were rehydrated with 20 mL of tap water for 48 h. Samples were shaken and rinsed, the supernatant were filtered through two stacked sieves (100 µm and 74 µm) [38]. The 74 µm sieve was used as a tool for fractionating the sample to facilitate sorting, rather than as a filter to discard material. Both the fraction retained in the 74 µm sieve and the entire volume that passed through it were examined under a stereoscopic microscope (45X, dark field illumination). The collection of specimens was made possible by permits granted by the Subsecretaría de Política Ambiental y Recursos Naturales-Dirección General de Vida Silvestre (SPARN/DGVS/06220/23). The transport and handling of the samples was carried out in strict compliance with Mexican legislation and the guidelines of the Ethics Committee of IBUNAM (CoÉtica-IBUNAM), guaranteeing confidentiality and respect for cultural and biological heritage.

**Table 1:** Sampling sites description. Sampling unit, substrate type (habitat), site/name, altitude zone with m asl, and landscape type of samples collected in the southwestern slope of the Iztaccihuatl volcano, from January to June in 2018.

Site/name	Elevation (m asl)	Sampling unit	Substrate	Landscape	Elevation zone
**S1** La Comunidad	2700	I-IV	Tree bark	*Pinus/Cupressus/Quercus* Forest	Alpine
**S2** Barranco Palomas	3030	V-X	Tree bark		
		XI	Soil	*Abies religiosa* forest	
**S3** Cañada Cueva del Negro	3278	XII-XIII	Soil		
		XIV	Tree bark		
**S4** Cañada Palo Rechino 1	3411	XV-XVI	Tree bark		
		XVII-XIX	Soil		
**S5** Cañada El Paraje	3498	XX	Tree bark		
		XXI-XXII	Soil		
**S6** Cañada Palo Rechino 2	3613	XXIII-XXVI	Soil		
		XXVII-XXVIII	Tree bark	*Pinus hartwegii* forest	
**S7** Paso de Cortés	3700	XXIX	Soil		
		XXX	Tree bark		
		XXXI	Soil		
		XXXII	Tree bark		
**S8** Subestación Eléctrica	3760	XXXIII-XXXV	Tree bark		
		XXXVI-XXXVII	Soil		
**S9** Refugio Altzomoni	3957	XXXVIII-XXXIX	Soil		
		XL-XLII	Rock		
**S10** Parador La Joya	4126	XLIII-XLVI	Rock		
		XLVII	Soil		
**S11** Primer Portillo	4235	XLVIII	Soil	alpine grassland	Nival
		XLIX-LII	Rock		
**S12** Segundo Portillo	4500	LIII-LIV	Soil	alpine tundra	
		LV-LVII	Rock		

All specimens for light microscopy were mounted individually on microscope slides in Heinze polyvinyl alcohol (PVA) medium. Observations were made using phase contrast microscopy (PCM) (ZEISS Axioskop with digital camera Axiocam ERC 55). The Morphospecies and species were identified using the taxonomic keys by Pilato and Binda [53], Claxton [54], Fontoura and Pilato [55], Kaczmarek et al. [56] Kaczmarek and Michalczyk [57]. The identification of *Macrobiotus* specimens was based on the oral cavity armature (OCA) proposed in Kaczmarek and Michalczyk [57], that identifications were based on the best available morphological evidence, based on high-resolution microscopy and compared multiple specimens per morphotype where possible to confirm diagnostic features. In some cases, species identification was not possible due to the small number of specimens and their poor condition. The genus abbreviations are used according to Perry et al. [58]. With the data of the identified specimens, abundance and presence-absence tables were elaborated (S1–S8 Tables), considering each of the 57 sampling units as independent tardigrade assemblies (*i.e.,* mosses of at least 300 cm^2^ of area growing on different substrates). To each sampling unit the data of the six replicates were summarized, thus we analyzed 57 tardigrade assemblies corresponding to three substrates, five landscapes, and two elevation zones (Table 1).

### Data analysis

The data analyses were carried out at three different ecological scales: 1) to determine the diversity of tardigrades in the habitat scale the assemblies were grouped based on the substrates where they were found (moss growing on bark, soil and rock), 2) to evaluate the tardigrade diversity on landscape scale, assemblies were pooled by landscape type (mixed, *A*. *religiosa*, *P*. *hartwegii* forests, alpine grassland and tundra), and 3) to identify the outcome at regional scale (elevation zones), tardigrades were pooled by elevation zones (alpine and nival zone).

#### Inventory completeness estimation.

The estimations of the completeness of the inventory and the potential number of species in each ecological scale were calculated using the sample coverage (SC) estimator, which is a measure of how representative the sample is of all community and is defined as the proportion of individuals in a community that belong to the species represented in the sample [59]. These estimates were carried out using the iNEXT version 1.3 program [60]. The dataset was randomized 100 times and compared with the observations [59]. To determine if there are significant differences between values estimated from resampling, A confidence interval (CI) of 95% was used. If the CI 95% overlaps between samples the estimates do not differ significantly under a significant value of 0.05. Conversely, if the CIs 95% do not overlap, it is concluded that the estimates differ significantly from each other [61]. An accumulation curve was calculated for each data set, also in iNEXT version 1.3 program [60].

#### Alpha diversity.

Hill’s numbers were used for the analysis of species diversity in each data set (S1–S4 Tables), specific richness or order 0, diversity of common species or order 1, and diversity of dominant species or order 2, according to Jost [62]. These estimates were carried out using the iNEXT version 1.3 program [60]. The analyses were performed with 100 randomizations and were extrapolated to twice the number of samples, using 95% interval confidence [59]. To compare the diversity values among the three ecological scales, the results were standardized to the same sample coverage (SC).

#### Beta diversity.

The taxonomic beta diversity was evaluated on each ecological scale. The taxonomic beta diversity was estimated with the Sorensen index (βSOR), analyzed in two components: the dissimilarity due to turnover (βSIM) and the dissimilarity due to differences in richness (nestedness) (βNES), both under the multiple-site approach [63]. Based on the natural consecutive distribution of landscape types in elevation gradient (under the pairwise approach), the beta diversity was measured (βsor = βsim + βnes), calculating the relative contribution of each component based on the incidence of the species [64] (S5–S6 Tables).

#### Assembly species by habitat, landscape and elevation zones.

To assess whether it is possible to recognize the local structure of tardigrade assembly at different ecological scales (*i.e.,* habitat, landscapes, elevation zones) a three two-way clustering analyses were performed, based on the absence-presence tables (S5–S7 Tables). In each scale, distance matrices were estimated with the Jaccard index and then a corresponding dendrogram with the simple clustering method, on PAST software ver 4.12 [63] were performed. The topology of the three trees was used to define the boundaries and composition of the local communities, including identifying exclusive and shared species among them. Species richness within these communities was visually represented using Venn diagrams. To evaluate the multidimensional spatial structure of assemblies among ecological scales a Principal Coordinate Analyses (PCoA) was performed on the presence-absence matrix. In the analysis, habitat, landscape, and altitude nival zone were used as OTU’s and species as attributes (S8 Table). The PCoA was computed in the PAST software ver 4.12 [65].

## Results

### General results

From the 342 moss replicates, 200 (58.5%) were positive, *i.e.,* contained tardigrades and/or eggs. The number of specimens per positive replicate was between 1–208, whereas the morphospecies number varied from 1 to 10.

A total of 1447 tardigrades and 30 eggs were found, and corresponding to six families, 13 genera, and 29 morphospecies, seven species, and 18 putative identifications (15 to *cf*. level, and three to *aff*. level) (Table 2). Macrobiotidae was the most abundant family (65.44%), followed by Hypsibiidae (23.91%) and Echiniscidae (8.22%) (Table 2). Hypsibiidae was the richest family, with four genera, five species and 13 putative identifications. Ramazzottidae, Doryphoribiidae and Milnesiidae, only recorded one genus each (Table 2). The most abundant morphospecies/species were *Minibiotus citlalium* (Macrobiotidae) (n = 476, and five eggs), *Mesobiotus* aff. *harmsworthi* (Macrobiotidae) (n = 216) (10 eggs), *Degmion nodulosus* (Hypsibiidae) (n = 157), *Macrobiotus hufelandi* OCA *lissostomus* (Macrobiotidae) (n = 110) (five eggs *Macrobiotus hufelandi* type) and, *Minibiotus sidereus* (Macrobiotidae) (n = 101) (10 eggs). The least abundant morphospecies/species were *Diphascon* cf. *claxtonae* (n = 1), *Dip.* cf. *faialense* (n = 1), *Dip.* cf. *victoriae* (n = 1), *Dip.* cf. *dastychiii* (n = 2), *Dip.* cf. *mitrense* (n = 2), *Hypsibius* 200 sp. nov. 3 (Hypsibiidae) (n = 3), *Hys.* 200 sp. nov. 4 (Hypsibiidae) (n = 3), *Hys.* 200 sp. nov. 1 (n = 4), and *Diphascon* cf. *pinguiforme* (n = 4), all belong to Hypsibiidae and *Ramazzottius* (Ramazzottidae) (n = 5).

**Table 2:** Tardigrada taxa presented in the southwestern slope of the Iztaccihuatl volcano. Presented by station number (S1-S12), elevation zone (2700-4500 m asl), and landscape type. In the bottom, the values of diversity (order 0, 1 and 2) are included, where no statistical differences between stations were observed.

Station	S1	S2	S3	S4	S5	S6	S7	S8	S9	S10	S11	S12	Total
**Altitude (m asl)**	**2700**	**3030**	**3278**	**3411**	**3498**	**3613**	**3700**	**3760**	**3957**	**4126**	**4235**	**4500**	
**landscape type**	**Mixed forest**		***Abies religiosa* forest**				***Pinus hartwegii* forest**				**a grassland**	**a tundra**	
**Echiniscidae**													
*Claxtonia* cf*. maucci*		1			6		8	58					73
*Pseudechiniscus* (*Pse.*) sp.	42				1		1			2			46
**Milnesiidae**													
*Milnesium* (*tardigradum*) sp.**[3-3, 3-3]**	1							4	2	2	1		10
**Hypsibiidae**													
*Diphascon* cf*. claxtonae*				1									1
*Dip.* cf*. dastychii*			2										2
*Dip.* cf. *faialense*	1												1
*Dip.* cf. *mitrense*	1	1											2
*Dip.* cf. *pingue*	4	5		1			4	6					20
*Dip.* cf*. pinguiforme*	4												4
*Dip.* cf*. victoriae*		1											1
*Hypsibius* cf. *microps*		1		1			3	2				1	8
*Hys.* cf. *pallidus*		14					3	7					24
*Hys.* 200 sp. nov. 1									3			1	4
*Hys.* 200 sp. nov. 2												6	6
*Hys.* 200 sp. nov. 3												3	3
*Hys.* 200 sp. nov. 4							2	1					3
*Hys.* 200 sp. nov. 5							1		2			6	9
*Hys.* cf. *pedrottii*	3	1							9			17	30
*Adropion scoticum*						4	40		9	6			59
*Adr. onorei*					5		1	2	12	2			22
*Degmion nodulosus*				2	22	1	78	54					157
**Ramazzottidae**													
*Ramazzottius* sp.					5								5
**Doryphoribiidae**													
*Doryphoribius* sp.	5	4			1								10
**Macrobiotidae**													
*Macrobiotus hufelandi OCA patagonicus*	3			1						5	1	2	12
*Mac. hufelandi OCA lissostomus*	49	2		53						2	2	2	110
*Mesobiotus* aff. *harmsworthi*	3		1	1	30	29	13		1	1	122	15	216
*Minibiotus sidereus*		5			12		20	64					101
*Min. citlalium*	1	91			83	2	63	229		1		6	476
*Paramacrobiotus* sp.	1		1	30									32
Taxa	13	11	3	8	9	4	13	10	7	8	4	10	29
Total abundance	118	126	4	90	165	36	237	427	38	21	126	59	1447
Hill’s number (q0)	13.53	10.95	4.03	11.37	7.02	3.77	9.73	6.00	6.84	8.25	1.22	9.82	
confidence interval	3.7-23.4	3.3-18.6	0.1-8.0	2.64-20.10	6.21- 7.83	1.61-5.93	9.63-12.53	4.76-7.24	3.45-10.23	3.05-13.44	0.00-3.30	5.47-14.17	
SC	0.96	0.96	0.88	0.96	0.96	0.96	0.96	0.96	0.96	0.96	0.96	0.96	
Hill’s number (q1)	4.89	3.01	3.95	2.87	4.22	1.94	5.65	3.70	5.14	6.86	1.09	6.78	
confidence interval	3.49-6.28	2.15-3.87	0.00-8.29	1.95-3.79	3.65-4.78	1.28-2.60	4.77-6.53	3.33-4.06	3.93-6.34	4.36-9.37	0.88-1.31	5.33-8.23	
SC	0.96	0.96	0.88	0.96	0.96	0.96	0.96	0.96	0.96	0.96	0.96	0.96	
Hill’s number (q2)	3.28	1.86	3.69	2.19	3.09	1.50	4.42	2.80	4.39	5.79	1.06	5.39	
confidence interval	2.62-3.94	1.51-2.20	0.00-9.22	1.81-2.58	2.54-3.65	1.03-1.96	3.90-4.95	2.50-3.10	3.42-5.37	3.77-7.82	0.98-1.13	4.11-6.68	
SC	0.96	0.96	0.88	0.96	0.96	0.96	0.96	0.96	0.96	0.96	0.96	0.96	

### Inventory completeness and alpha diversity

In total, thirty-three species were estimated; the 29 observed taxa correspond to 87% completeness of the inventory.

#### Tardigrade assemblies at the habitat scale.

**Fig 2:**
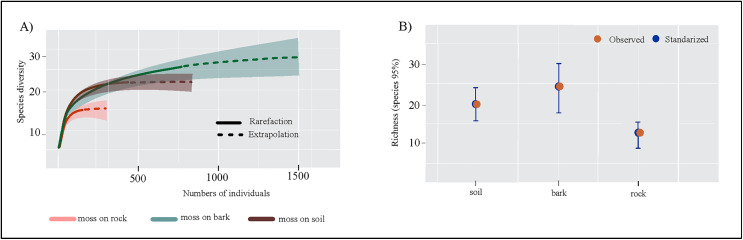
Richness and diversity of tardigrades by habitat type, from Iztaccíhuatl volcano. **A)** accumulation curve; **B)** Observed and standardized values of species richness (q0) for each habitat type sampled of the sampled sites. For standardized diversity values, error bars are 95% conﬁdence intervals (CI).

In the three types of habitat, the sample coverage was 0.99 SC with normalized values (Table 3). Moss on bark and soil recorded the highest abundance (N_bark_ = 830; N_soil_ = 454) and richness (S_bark_ = 24; S_soil_ = 20) of tardigrada taxa, which in turn represent 89% and 99% of the inventoried taxa, respectively (Fig 2A and 2B) (Table 3). The mosses on rocks presented the lowest abundance (N_rock_ = 158) and richness (S_rock_ = 12), and it inventoried the 98% of the taxa present in this habitat type (Fig 2A and 2B and Table 3). Mosses on bark tree and mosses on soil show a similar community structure, where the values of richness (q0) and diversity of common species (q1) do not show statistically significant differences, but not in diversity of order 2 where statistical differences between the three habitat were found (Table 3).

**Table 3:** Alpha diversity by the habitat scale. Sample number, sample coverage (SC), abundance (N), richness (S), diversity order 0 (q0), diversity order 1 (q1), diversity order 2 (q2), and inventory completeness of each data set habitat, landscape and elevation).

		sample number	SC	N	S	q0	q1	q2	Inventory Completeness
Habitat type	bark	21	.99	830	24	24 (15-38)	8.6 (8-9.3)	6 (5.4-6.3)	89.23%
	soil	21	.99	454	20	20.24 (16-25)	7.4 (6.4-8.3)	4.2 (3.6-4.7)	98.79%
	rock	14	.99	158	12	12.24 (8-16)	3.8 (3-4.5)	2 (1.7-2.5)	98.12%
Landscape type	Mixed forest	11	.97	245	19	19 (3-35)	7 (5.4-8)	4.5 (3.7-5.2)	75.75%
	*Abies religiosa* forest	11	.97	254	15	15 (2-28)	6.7 (5.6-7.8)	5.1 (4.4-5.9)	74.64%
	*Pinus hartwegii* forest	25	.99	760	19	19 (15-22)	6.83 (6.3-7.2)	4.7 (4.2-5.1)	97.77%
	Alpine grassland	5	.98	126	4	4 (1-7)	1.0 (0.8-1.1)	1.0 (0.9-1.0)	76.04%
	Alpine tundra	5	.96	59	10	10 (7-13)	6.4 (5.0-7.7)	5.1 (3.8-6.5)	92.16%
Elevation zone	Alpine zone 2,700−3,957 m	47	.99	1236	27	27 (21-32)	9.2 (8.6-9.8)	5.4 (4.9-5.9)	92.27%
	Nival zone 4,126−4,500 m	10	.99	206	14	14 (10-17)	4 (3.2-4.8)	2.1 (1.7-2.5)	94.33%

#### Tardigrade assemblies at landscape scale.

**Fig 3:**
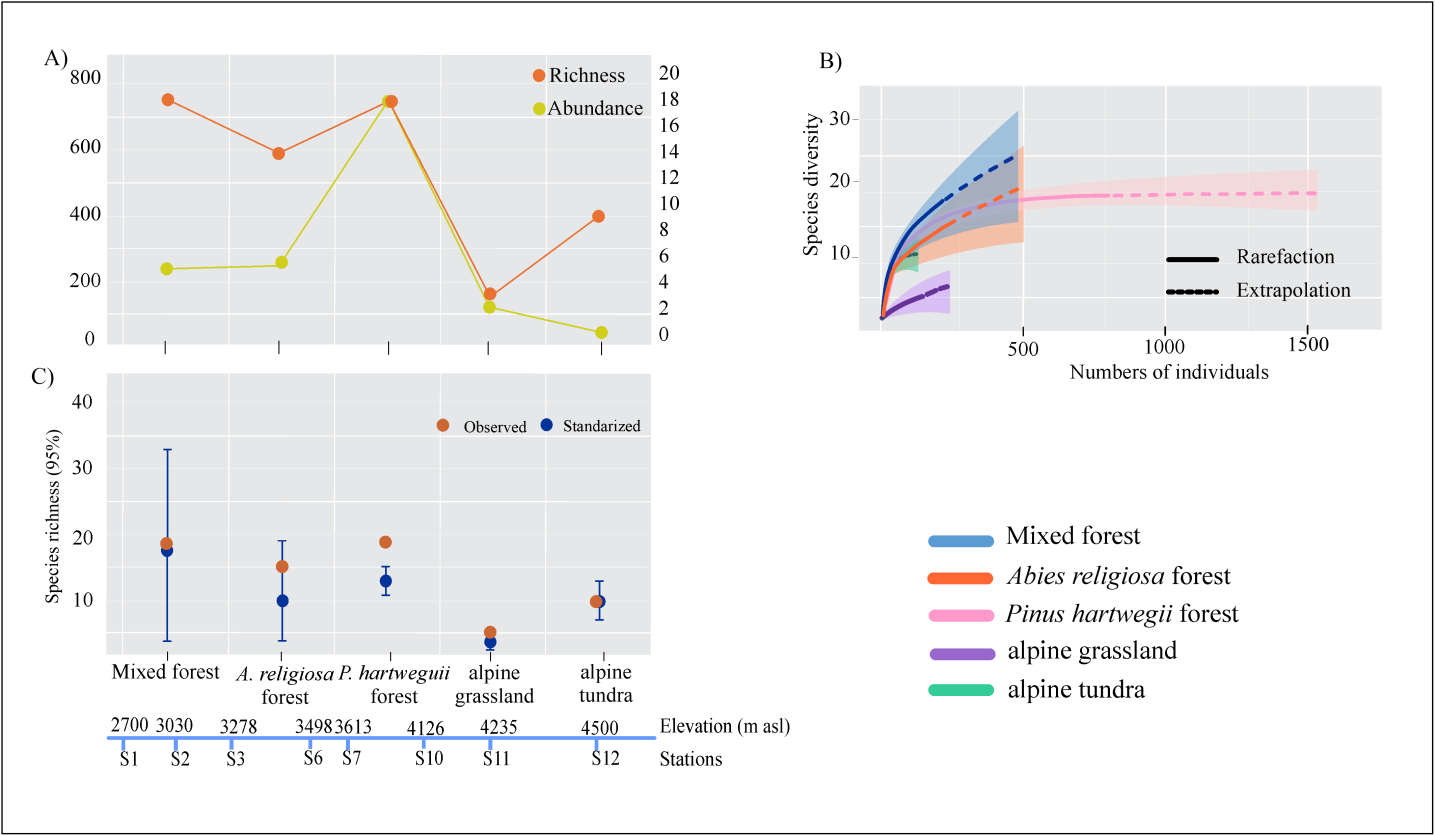
Richness and diversity of tardigrades by landscape type, from Iztaccihuatl volcano. **A)** Richness and Abundance distribution; **B)** Accumulation curve; **C)** Observed and standardized values of species richness for each sampled landscape type on sampled sites. For standardized diversity values, error bars are 95% conﬁdence intervals (CI).

The *Pinus hartwegii* forest recorded the highest abundance (N = 760) and share with mixed forest the highest richness (S=19) (Table 3 and Fig 3A), in mixed forest these 19 taxa represented the 75% of the tardigrade fauna, while in *Pinus hartwegii* forest it represented the 97% of the tardigrade taxa (Table 3 and Fig 3B). The sample coverage presented values ranged from 0.96-0.99, standardized SC to 0.96 shows higher richness and diversity in coniferous forest (mixed forest, *Abies religiosa* and *Pinus hartwegii* forest), than in alpine grassland and tundra, but according to the confidence intervals of species richness (q0), there were no statistical difference of richness and diversity through landscapes (Table 3 and Fig 3C). The observed and estimated diversity at 1 and 2 orders presented statistical differences, between coniferous forest and alpine tundra with higher values than alpine grassland (Table 3).

The sampling effort allowed us to recover 29 taxa and corresponding to 87% of the total tardigrade species presented in the study area. To reach the asymptote, the collecting effort should be concentrated in the mixed and *Abies religiosa* forest (2700-3498m asl), as well as in the alpine grassland (4126 m asl), where the species recorded were ≤ 75%, and in mosses growing on tree bark ≤ 89% (Table 3). Regarding the landscape type, the difference between observed *vs* estimated species lies in the difference in the number of stations, and the number of samples collected on each landscape type (Table 2). In the mixed forest and *Abies religiosa* forest, 11 samples of each were collected, while in *Pinus hartwegii* forest, 25 samples were collected and 97% of the species were recorded, more than twice as many samples as in other landscape types. In the study area the *Pinus hartwegii* forest domanates in spatial extent, which is reflected in the number of stations (Table 2), and in the availability of moss for collection.

#### Tardigrade assemblies at elevation scale.

**Fig 4:**
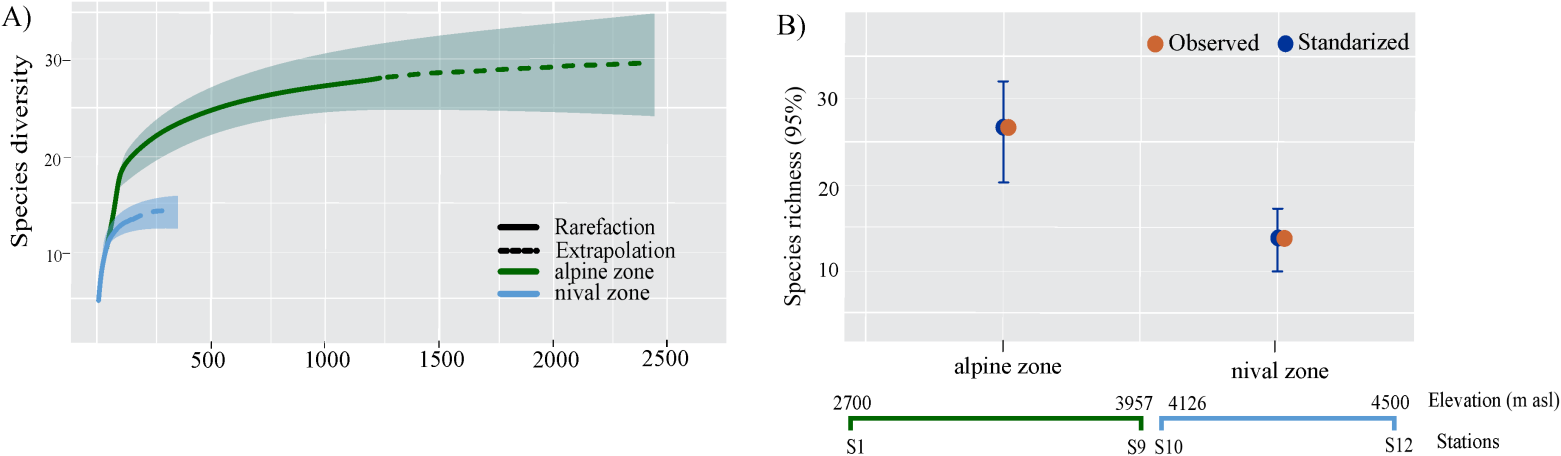
Richness and diversity of tardigrades by elevation zone, from Iztaccihuatl volcano. **A)** accumulation curve; **B)** Observed and standardized values of species richness for each elevation zone. For standardized diversity values, error bars are 95% conﬁdence intervals (CI).

The sample coverage (SC) of each interval elevation was high, with values of 0.99 (Table 3). The alpine zone elevation (2700–3957 m asl) had greater abundance (N = 1241), and richness (S=27), and 92% of the taxa presented were recorded in this zone (Fig 4A). In the nival zone 206 individuals were observed, corresponding to 14 taxa (Table 3 and Fig 4A). According to the confidence intervals, there was a statistical difference between richness and diversity at both elevation zones (Fig 4B).

### Beta diversity

#### Tardigrade assemblies at the habitat scale.

The taxonomic beta diversity (total dissimilarity) among the three habitat types analyzed was 45.83% (βSOR) and is mainly due to species turnover (βSIM 27.77%) with a low nestedness contribution (βNES = 18.05%). Total dissimilarity was low, with a value of <50%, and was explained by species turnover. Pairwise analysis showed that the greatest dissimilarity is observed between the communities found in tree bark and rock (βSOR = 50%), while the least dissimilarity was between the rock and soil habitats (βSOR = 31.20%), (and the dissimilarity between these two habitatswas explained by loss of species) (Table 4).

**Table 4:** Beta diversity by the habitat scale. Total taxonomic beta diversity (total dissimilarity [βsor]) as the sum of its components (turnover [βsim] and nestedness [βnes]) for the Tardigrada community along a habitat type in the Iztaccihuatl Volcano.

Taxonomic beta Diversity			
Pair habitat types	βsim	βnes	βsor
tree bark *vs* rocks	25%	25%	50%
tree bark *vs* soil	30%	6.30%	36.30%
rocks *vs* soil	8.30%	22.90%	31.20%

#### Tardigrade assemblies at landscape scale.

The taxonomic beta diversity (total dissimilarity) among the five landscape types was 64.15% (βSOR) and was explained by species turnover (βSIM = 41.53%), with a low nestedness contribution (βNES = 22.61%). Among the coniferous forest (mixed, *Abies religiosa* and *Pinus hartwegii*), the beta diversity was mainly due to the turnover of species, meanwhile, in alpine grassland and tundra is mainly due to the nestedness (Table 5).

**Table 5:** Beta diversity by landscape scale. Total taxonomic beta diversity (total dissimilarity [βsor]) as the sum of its components (turnover [βsim] and nestedness [βnes]) for the Tardigrada community along a landscape type through elevational gradient of the Iztaccihuatl Volcano.

Taxonomic beta Diversity			
Pair landscape types	βsim	βnes	βsor
Mixed forest vs *Abies religiosa* forest	26.66%	8.60%	35.20%
*Abies religiosa* forest vs *Pinus hartwegii* forest	26.66%	10.40%	37.0%
*Pinus hartwegii* forest vs alpine grassland	0	66.60%	66.60%
Alpine grassland vs alpine tundra	25%	32.14%	57.10%

#### Tardigrade assemblies at elevation scale.

The taxonomic beta diversity (total dissimilarity) among the two elevation zones was 41.46% (βSOR) and was mainly due to species nestedness (βNES = 27.17%) with a low turnover contribution (βSIM = 14.28%).

### Community structure and composition by habitat, landscape and elevation zone

The obtained dendrograms of the three ecological scales showed a good consistency between the original distance matrix and the resulting topology, with values of co-phenetic correlation index of, r_habitat_  = 0.73, r_landscape_ = 0.93, and r_elevation_ = 0.91 respectively, and the recovered clusters were supported by values from 70−60% in most cases.

#### Habitat scale.

**Fig 5:**
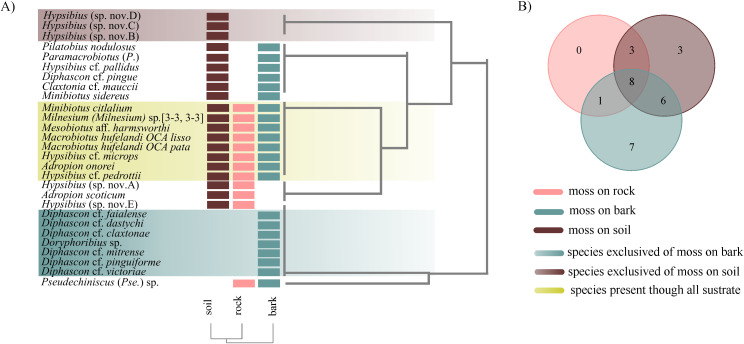
Local communities of tardigrades respect to habitat type. **A)** Two-way cluster analysis of pool species community based on presence-absence matrix and Jaccard index; **B)** Venn diagram among local communities recognized in two-way cluster analysis. Mosses on soil and rocks harbor the most similar assemblies sharing 11 species at this scale.

The topology and Venn diagram showed that eight taxa were recorded in all habitat types analyzed (mosses on rocks, soil and tree bark): *Minibiotus citlalium*, *Milnesium* (*tardigradum*) sp. [3-3, 3-3], *Mesobiotus* aff. *harmsworthi*, *Macrobiotus hufelandi* OCA *lissostomus* and *Mac. hufelandi* OCA *patagonicus*, *Hypsibius* cf. *microps*, *Adropion onorei*, *Hypsibius* cf. *pedrottii* (Fig 5A and 5B). The mosses on tree bark presented 21 of the 29 recorded taxa, besides nine taxa were exclusive of this habitat: *Diphascon* cf*. faialense, Dip.* cf*. dastychii, Dip.* cf*. claxtonae, Doryphoribius* sp*., Diphascon* cf. *mitrense, Dip.* cf*. pinguiforme, Dip.* cf*. victoriae,* and *Hypsibius* (Fig 5A and 5B). Three taxa were exclusive of mosses on soil: *Hypsibius* 210 (sp. nov. 2), *Hys.* 210 (sp. nov. 3), and *Hys.* 210 (sp. nov. 4). The mosses on rocks did not present an exclusive taxon, but rather share three taxa with mosses on soil: *Hypsibius* 210 (sp. nov. 1), *Hys.* 210 (sp. nov. 5), and *Adropion scoticum.*

#### Landscape scale.

**Fig 6:**
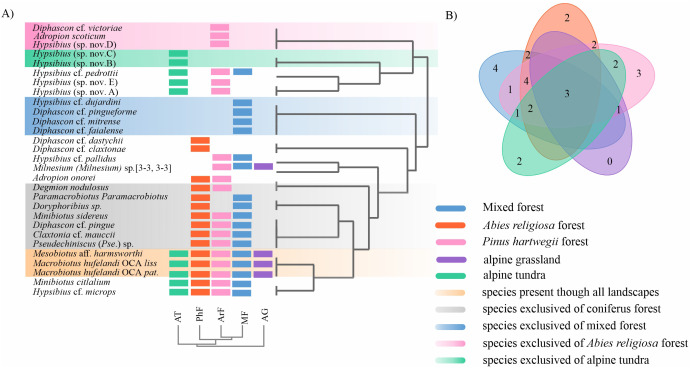
Local communities of tardigrades respect landscape type. **A)** Two-way cluster analysis of pool species community based on presence-absence matrix and Jaccard index; **B)** Venn diagram among local communities recognized in two-way cluster analysis.

The dendrogram topology and Venn diagram showed that, *Mesobiotus* aff. *harmsworthi*, *Macrobiotus hufelandi* OCA *lissostomus* and *Mac. hufelandi* OCA *patagonicus* were recorded in the five landscape types (mixed, *Abies religiosa* and *Pinus hartwegii* forest, alpine grassland and tundra). The coniferous forest (mixed, *Abies religiosa* and *Pinus hartwegii* forest, 2700-4126 m asl) presents a consistently composition community, together they present 28 out of the 29 recorded taxa. Also, nine taxa were exclusive of coniferous forest: *Adropion onorei*, *Degmion nodulosus*, *Paramacrobiotus* sp*.*, *Doryphoribius sp.*, *Minibiotus sidereus*, *Diphascon* cf. *pingue*, *Claxtonia cf. maucci*, and *Pseudechiniscus* (*Pse.*) sp*.* (Fig 6A and 6B). Based on species exclusive to each landscape type, within the coniferous forest, local communities were recognized, five taxa were exclusive of mixed forest (2700-3030m asl): *Hypsibius dujardini*, *Diphascon* cf. *pinguiforme*, *Dip.* cf. *mitrense*, and *Dip.* cf. *faialense*. Three taxa were exclusive of *Abies religiosa* forest (3278-3498m asl): *Diphascon* cf. *victoriae*, *Adropion scoticum*, and *Hypsibius* (sp. nov. 4), and two taxa were exclusive of *Pinus hartwegii* forest (3613-4126 m): *Diphascon* cf. *dastychiii* and *Dip.* cf. *claxtonae* (Fig 6A and 6B).

The alpine grassland has no exclusive taxa, while in alpine tundra two taxa were exclusive: *Hypsibius* (sp. nov. 2) and *Hys.* (sp. nov. 3) (Fig 6A and 6B).

#### Elevation zones.

**Fig 7:**
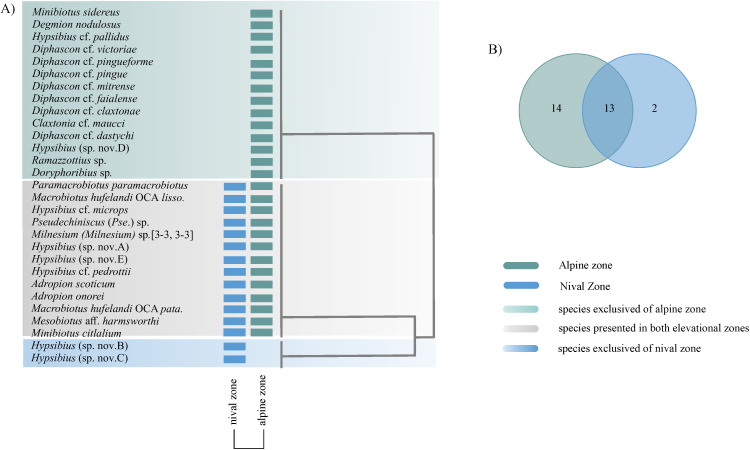
Local communities of tardigrades respect elevation zones. **A)** Two-way cluster analysis of pool species community based on presence-absence matrix and Jaccard index; **B)** Venn diagram among local communities recognized in two-way cluster analysis.

The topology and Venn diagram showed that thirteen taxa were recorded in both elevation zones, alpine and nival, from 2700-4500 m asl (Fig 7A and 7B). In alpine zone 27 of the 29 recorded taxa, besides 14 taxa were exclusive (Fig 7A and 7B). The richness of the nival zone is generally a subset of the alpine zone (Fig 7A). Two taxa were exclusive of the nival zone: *Hypsibius* 210 (sp. nov. 2), *Hys* 210 (sp. nov. 3).

### Community structure by habitat, landscape and elevation zones

**Fig 8:**
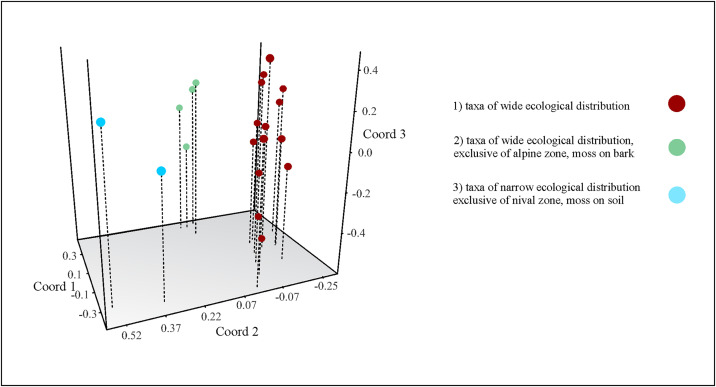
Scatter plot corresponding to principal coordinate analyses (PCoA) of tardigrade community data from Iztaccihuatl Volcano, based on presence-absence matrix and Jaccard index.

Finally, three different patterns of habitat preference were recognized with the PCoA: 1) species/taxa that were widely recorded in the study area (*Adropion onorei*, *Adr*. *scoticum*, *Diphascon cf. victoriae*, *Dip*. cf. *pingue*, *Claxtonia* cf. *maucci*, *Hypsibius* sp. nov. 1, *Hys*. sp. nov. 4, *Hys*. sp. nov. 5, *Hys*. cf. *microps*, *Hys*. cf. *pedrottii, Hys*. cf. *pallidus, Degmion nodulosus, Macrobiotus hufelandi* OCA *lissostomus, Mac. hufelandi* OCA *patagonicus, Mesobiotus* aff. *harmsworthi*, *Minibiotus sidereus*, *Min*. *citlalium*, and *Pseudechiniscus* (*Pse.*)

sp.). The next two patterns include taxa with narrow ecological valence, 2) Species/taxa which were exclusive of alpine zone, mixed and *Abies religiosa* forest (2700-3500m asl) and only were found in moss growing on bark (*Diphascon* cf. *claxtonae*, *Dip*. cf. *dastychii*, *Dip.* cf. *faialense*, *Dip*. cf. *mitrense*, *Dip*. cf. *pinguiforme*, *Paramacrobiotus* sp*.*, and *Doryphoribius* sp.). 3) Finally, two taxa were exclusive to nival zone, in alpine tundra, were found in moss growing on soil (*Hypsibius* 210 sp. nov. 2, *Hys*. 210 sp. nov. 3) (Table 2 and Fig 8).

## Discussion

### Inventory and alpha diversity

A typical moss sample yields between 5 and 10 tardigrade species, although this number can range from a single species to more than 15, depending on environmental conditions and sampling methodology [8,15,66,67]. Richer assemblages are often found in tropical or well-preserved habitats, where environmental heterogeneity and stability support greater species diversity [68]. In our study, each sampling unit (*i.e.,* moss sample) contained an average of eight species, with a maximum of 13 species per sample, placing our findings within the expected range for diverse and structurally complex moss communities.

### Tardigrade assemblies at the habitat scale

Our results showed that mosses growing on bark and soil supported the highest tardigrade abundances (N_bark_ = 830; N_soil_ = 454) and species richness (S_bark_ = 24; S_soil_ = 20), representing 89% and 99% of the total recorded taxa, respectively (Fig 2A and 2B and Table 3). These findings highlight the role of habitat-driven microhabitat differences in shaping tardigrade community composition at fine spatial scales [13–15]. Notably, mosses on bark hosted seven exclusive species, while mosses on soil supported three exclusive taxa—mainly undescribed species of *Hypsibius*—suggesting distinct environmental conditions that promote species-specific associations. These patterns are consistent with previous studies showing that, the habitat on which mosses grow significantly influences tardigrade diversity and community structure [34,69–71]. Terricolous mosses (on soil) typically offer stable moisture levels, rich organic matter, and greater structural complexity [72], and corticolous mosses (on tree bark) benefit from vertical microclimatic gradients and canopy influences, providing varied ecological niches across tree trunks [15,69–71]. Our findings reinforce the importance of habitat heterogeneity in supporting diverse and structured tardigrade assemblages in moss microhabitats.

Most ecological studies report that between 3 and 10 species can be shared across soil, bark, and rock substrates within a given landscape, depending on sampling intensity and environmental variability [15,73]. Among the 29 tardigrade taxa recorded in Iztaccihuatl volcano, eight species were found across all three moss habitats—moss on rocks, soil, and tree bark—indicating a group of broadly adaptable taxa capable of thriving in diverse microhabitats. These generalist species include *Minibiotus citlalium*, *Milnesium* (*tardigradum*) sp. [3-3, 3-3], and representatives of the *Macrobiotus* and *Hypsibius* genera. Our results also indicate that soil and rock habitat harbor the most similar tardigrade assemblages, sharing 11 species, because they share more environmental characteristics—such as ground-level microclimate, exposure, and moisture dynamics, while moss on bark habitat tend to support more distinct communities, likely due to their vertical position, increased microclimatic variability, and canopy effects [69,73]. Bartels and Nelson [15] similarly reported that soil and rock mosses are dominated by generalist taxa, especially in temperate and alpine regions, whereas bark mosses host more specialized or vertically distributed species. These highlights how habitat, and in this case substrate-driven microclimatic stability and habitat connectivity shape tardigrade community composition at fine ecological scales.

### Tardigrade assemblies at the landscape scale

Tardigrade surveys typically report between 30 and over 100 species, depending largely on habitat heterogeneity, elevation range, and sampling intensity [14,21]. Tardigrade species richness tends to be highest in tropical and temperate forested regions, and lower in polar or arid environments where harsher conditions limit diversity [6,14,15,67]. The Iztaccíhuatl volcano harbored a total of 29 tardigrade taxa corresponding to 87% completeness of the inventory, a figure consistent with expectations for temperate montane systems with moderate sampling effort (only mosses). For comparison in other mountains, Bartels and Nelson [15] recorded 42 species in a temperate forest through extensive, long-term sampling across various microhabitats (mosses, lichens, soil, leaf litter), and Kaczmarek et al. [74] reported 38 species from a *Pinus* forest through similarly diverse microhabitat sampling. While Kaczmarek et al. [14] reported 64 species through different tropical forests. These comparisons suggest that the tardigrade richness on Iztaccíhuatl volcano reflects both the elevational range and ecological complexity of the area, though further sampling across additional microhabitats may reveal higher species richness.

The coniferous forest—comprising mixed, *Abies religiosa* and *Pinus hartwegii*—presented the highest values of richness and abundance, confirming what was reported by Guil et al. [30], Richardson et al. [31], and Nelson et al. [32], who report that in dense forests there is a greater richness of tardigrades, related to abundant vegetation, high humidity with a large amount of organic matter, and the occurrence of other invertebrates as a food resource. Besides high taxonomic richness, Guil and Sanchez-Moreno [11] found high functional tardigrade diversity in areas with broad leaves as well, associated with dense forests and humid conditions [13,30], promoting more complex trophic webs and biotic relationships, because of their antiquity and stability in the time.

### Tardigrade assemblies at regional scale

The highest richness and diversity were recorded in the alpine zone (2,700−3,957 m asl), showing that these community attributes decrease as elevation increases, which is consistent with Guidetti et al. [8] in an analyzed gradient 1,200−1,700 m asl, Beasley [26] (1,050−3,560 m asl) and Surmacz et al. [75] (306−2,486 m asl). Other studies have shown maximum richness at intermediate elevation (e.g., 1,500–3,000 m), in a wider gradient from 0 to 4,000 m asl [13,14,24,25,76]. In general, depending on the scale of study, we could observe different trends in abundance and richness.

Based on the estimated richness the maximum species richness in the study area would be observed at 2,700−3,030 m asl (Table 3). The exploration of arthropods diversity in Iztaccíhuatl volcano, Cutz-Pool et al. [77,78] have reported other animal groups, as Acari, Collembola, Coleoptera, Psocoptera, Thysanoptera, Hemiptera, Hymenoptera, Arachnida, Myriapoda, and Oligochaeta, have shown a maximum abundance and richness from 2,750-3,020 m asl. Thus, regardless of elevation range, the abundance and richness of tardigrades and arthropods in the study area, is greater at intermediate elevations, particularly from 2,700−3,030m asl.

When analyzing diversity indices (q0, q1, q2) across stations located at different elevations, the diversity values of common and dominant species appear to be higher above 3,000 m asl, likely influenced by the greater abundance of tardigrades found at these elevations. Similar patterns have been reported in other studies, where tardigrade presence increases at elevations above 3,000 m, primarily due to species that are specialized for high-elevation environments [14,21].

### Scale interaction

The structure of tardigrade communities on the Iztaccíhuatl volcano exhibits a clear pattern of scale-dependent ecological variation, where each hierarchical level—from the habitat (substrates), through the landscape (vegetation types), to the regional scale (elevation zones)—contributes differently to the beta diversity. At the habitat scale, taxonomic dissimilarity among the three moss types analyzed (on tree bark, soil, and rocks) was mainly due to moderate (βSOR = 45.83%), primarily driven by species turnover (βSIM = 27.77%) and to a lesser extent by nestedness (βNES = 18.05%). This pattern suggests that although some taxa are habitat specialists, a set of generalist species is broadly distributed across habitats, which reduces total dissimilarity. A similar pattern was found in Kaczmarek et al. [14], when we are calculating the beta diversity of the data obtained between the same habitat of the present study—rocks, soil, bark—, but in more than one bryophyte type, finding as well, a low dissimilarity, below 50% and this given mainly by turnover.

At the landscape scale, the dissimilarity is above 60% and is given mainly by the substitution of species in the different landscapes, which suggests that there are species adapted to each of the landscapes [11].

The coniferous forests—comprising mixed forest, *Abies religiosa* forest, and *Pinus hartwegii* forest—acted as centers of both richness and compositional cohesion, because they share a greater number of species among them, low dissimilarity. Of the 29 taxa recorded, 27 were present in at least one of these three forest types, indicating high community connectivity within this coniferous complex. Based on other studies, [14,79], the coniferous forests represents tardigrada species reservoirs, harbor higher richness and diversity of tardigrada species, respect to other mountain landscapes.

However, in the transition from coniferous forests to grassland and alpine tundra, the dissimilarity increases, which was explained in greater proportion by the loss and gain of species in these landscapes, A similar pattern was found by [22], through their analysis, the glacial foreland—initial successional stage—recorded contrastingly lower richness than dry and wet tundra, both are a late stages of successional development—.

At the regional scale, defined by altitudinal zones, pronounced shifts in species composition were observed. In general, the species dissimilarity was low, so the overall composition is homogeneous, and the few differences that were observed were mainly due to the gain and loss of species. Different environmental conditions have been registered between both altitudinal zones, particularly, the nival zone—characterized by higher elevation and more extreme conditions—exhibited a reduced richness, and the community present in this zone was a subset of the alpine community.

Taken together, these findings confirm that tardigrade beta diversity increases with ecological scale, from moderate species turnover among habitats to more structured differentiation among landscape types, as discovered by Surmacz et al. [75], where altitude and geographic location determine species turnover, while microhabitat characteristics governed community structure. The coexistence of generalist and specialist species across these environmental gradients highlights the interaction between habitat heterogeneity, underscoring the importance of multiscale approaches in understanding and conserving microscopic biodiversity [80].

## Conclusions

The multiscale analysis of tardigrade communities showed that species diversity and composition are strongly determined by hierarchical factors ranging from habitat type to elevational gradients. The preference for mosses on bark and the concentration of richness in coniferous forests and alpine zones highlight the importance of these habitats for conservation. Likewise, the identification of exclusive species such as *Diphascon* spp., and *Hypsibius* sp. nov. 2 and 3 are exclusive to moss on tree bark and to nival zone respectively -including potentially new taxa for science- underlines the need to protect both high mountain environments and transitional ecosystems, given their role in maintaining the connectivity and diversity of the tardigrade fauna.

## Supporting information

S1 Table Abundance matrix used for the alpha diversity analysis across different sampling sites (Hill numbers: 0, 1, and 2), performed using the iNext program with a 95% confidence interval and 50 bootstraps. (DOCX)

S2 Table Abundance matrix of taxa used for alpha diversity analysis and accumulation curves at different habitats types, (Hill numbers: 0, 1, and 2), performed using the iNext program with a 95% confidence interval and 50 bootstraps. (DOCX)

S3 Table Abundance matrix of taxa used for alpha diversity analysis and accumulation curves at different landscape types, (Hill numbers: 0, 1, and 2), performed using the iNext program with a 95% confidence interval and 50 bootstraps. (DOCX)

S4 Table Abundance matrix of taxa used for alpha diversity analysis and accumulation curves in the alpine and nival zones (Hill numbers: 0, 1, and 2), performed using the iNext program with a 95% confidence interval and 50 bootstraps. (DOCX)

S5 Table Presence-absence matrix used for beta diversity and two-way cluster analysis by habitat scale, performed using the R program and Past Program. Beta diversity was performed with Total taxonomic beta diversity (total dissimilarity [βsor]) as the sum of its components (turnover [βsim] and nestedness [βnes]).
(DOCX)

S6 Table Presence, absence matrix used for beta diversity and two-way cluster analysis by landscape scale, performed using the R program and Past Program. Beta diversity was performed with Total taxonomic beta diversity (total dissimilarity [βsor]) as the sum of its components (turnover [βsim] and nestedness [βnes]).
(DOCX)

S7 Table Presence, absence matrix used for the two-way cluster analysis by elevation zones. This analysis was performed in Past software.
(DOCX)

S8 Table Presence-absence matrix used for Principal Coordinate Analyses (PCoA) analyses, using Jaccard index in Past software. (DOCX)
