# Supplementary material for: Beyond Ubiquity: Scale-dependent patterns of tardigrade diversity on the Iztaccíhuatl volcano
Source: PLoS One. 2026 Mar 4;21(3):e0343098. doi: 10.1371/journal.pone.0343098 (PMC12959721; doi:10.1371/journal.pone.0343098)
Supplement: S1 Table — (DOCX) [file pone.0343098.s001.docx]

Supporting Information

**Beyond Ubiquity: Scale-dependent patterns of tardigrade diversity on the Iztaccíhuatl volcano,**

Alba Dueñas-Cedillo ^1 #a^, Francisco Armendáriz-Toledano ^2¶*^, Rodolfo Cancino-López ^3^, Jazmín García-Román ^1 #a^, Enrico Alejandro Ruiz ^1¶*^

S1 Table. Abundance matrix used for the alpha diversity analysis across different sampling sites (Hill numbers: 0, 1, and 2), performed using the iNext program with a 95% confidence interval and 50 bootstraps.

| **2700** | **3030** | **3278** | **3411** | **3613** | **3700** | **3957** | **4007** | **4126** | **4235** | **4398** | **4500** |
| --- | --- | --- | --- | --- | --- | --- | --- | --- | --- | --- | --- |
| 49 | 91 | 2 | 53 | 29 | 78 | 9 | 229 | 6 | 122 | 83 | 17 |
| 42 | 14 | 1 | 30 | 4 | 63 | 9 | 64 | 5 | 2 | 30 | 15 |
| 5 | 5 | 1 | 2 | 2 | 40 | 3 | 58 | 2 | 1 | 22 | 6 |
| 4 | 5 | 0 | 1 | 1 | 20 | 2 | 54 | 2 | 1 | 12 | 6 |
| 4 | 4 | 0 | 1 | 0 | 13 | 2 | 7 | 2 | 0 | 6 | 6 |
| 3 | 2 | 0 | 1 | 0 | 8 | 1 | 6 | 1 | 0 | 1 | 3 |
| 3 | 1 | 0 | 1 | 0 | 4 | 0 | 4 | 1 | 0 | 1 | 2 |
| 3 | 1 | 0 | 1 | 0 | 3 | 0 | 2 | 0 | 0 | 0 | 2 |
| 1 | 1 | 0 | 0 | 0 | 3 | 0 | 1 | 0 | 0 | 0 | 1 |
| 1 | 1 | 0 | 0 | 0 | 2 | 0 | 1 | 0 | 0 | 0 | 1 |
| 1 | 1 | 0 | 0 | 0 | 1 | 0 | 0 | 0 | 0 | 0 | 0 |
| 1 | 1 | 0 | 0 | 0 | 1 | 0 | 0 | 0 | 0 | 0 | 0 |
| 1 | 0 | 0 | 0 | 0 | 0 | 0 | 0 | 0 | 0 | 0 | 0 |
| 0 | 0 | 0 | 0 | 0 | 0 | 0 | 0 | 0 | 0 | 0 | 0 |
| 0 | 0 | 0 | 0 | 0 | 0 | 0 | 0 | 0 | 0 | 0 | 0 |
| 0 | 0 | 0 | 0 | 0 | 0 | 0 | 0 | 0 | 0 | 0 | 0 |
| 0 | 0 | 0 | 0 | 0 | 0 | 0 | 0 | 0 | 0 | 0 | 0 |
| 0 | 0 | 0 | 0 | 0 | 0 | 0 | 0 | 0 | 0 | 0 | 0 |
| 0 | 0 | 0 | 0 | 0 | 0 | 0 | 0 | 0 | 0 | 0 | 0 |
| 0 | 0 | 0 | 0 | 0 | 0 | 0 | 0 | 0 | 0 | 0 | 0 |
| 0 | 0 | 0 | 0 | 0 | 0 | 0 | 0 | 0 | 0 | 0 | 0 |
| 0 | 0 | 0 | 0 | 0 | 0 | 0 | 0 | 0 | 0 | 0 | 0 |
| 0 | 0 | 0 | 0 | 0 | 0 | 0 | 0 | 0 | 0 | 0 | 0 |
| 0 | 0 | 0 | 0 | 0 | 0 | 0 | 0 | 0 | 0 | 0 | 0 |
| 0 | 0 | 0 | 0 | 0 | 0 | 0 | 0 | 0 | 0 | 0 | 0 |
| 0 | 0 | 0 | 0 | 0 | 0 | 0 | 0 | 0 | 0 | 0 | 0 |
| 0 | 0 | 0 | 0 | 0 | 0 | 0 | 0 | 0 | 0 | 0 | 0 |
| 0 | 0 | 0 | 0 | 0 | 0 | 0 | 0 | 0 | 0 | 0 | 0 |
| 0 | 0 | 0 | 0 | 0 | 0 | 0 | 0 | 0 | 0 | 0 | 0 |
