# Supplementary material for: Beyond Ubiquity: Scale-dependent patterns of tardigrade diversity on the Iztaccíhuatl volcano
Source: PLoS One. 2026 Mar 4;21(3):e0343098. doi: 10.1371/journal.pone.0343098 (PMC12959721; doi:10.1371/journal.pone.0343098)
Supplement: S2 Table — (DOCX) [file pone.0343098.s002.docx]

Supporting Information

**Beyond Ubiquity: Scale-dependent patterns of tardigrade diversity on the Iztaccíhuatl volcano,**

Alba Dueñas-Cedillo ^1 #a^, Francisco Armendáriz-Toledano ^2¶*^, Rodolfo Cancino-López ^3^, Jazmín García-Román ^1 #a^, Enrico Alejandro Ruiz ^1¶*^

S2 Table. Abundance matrix of taxa used for alpha diversity analysis and species accumulation curves at different substrate types, (Hill numbers: 0, 1, and 2), performed using the iNext program with a 95% confidence interval and 50 bootstraps.

| bark | soil | rock |
| --- | --- | --- |
| 270 | 199 | 108 |
| 129 | 71 | 9 |
| 100 | 50 | 7 |
| 95 | 28 | 7 |
| 62 | 20 | 6 |
| 44 | 12 | 6 |
| 37 | 11 | 4 |
| 26 | 8 | 3 |
| 16 | 8 | 3 |
| 12 | 7 | 2 |
| 10 | 6 | 2 |
| 5 | 6 | 1 |
| 4 | 6 | 0 |
| 4 | 6 | 0 |
| 4 | 5 | 0 |
| 3 | 3 | 0 |
| 2 | 3 | 0 |
| 2 | 2 | 0 |
| 2 | 2 | 0 |
| 1 | 1 | 0 |
| 1 | 0 | 0 |
| 1 | 0 | 0 |
| 1 | 0 | 0 |
| 1 | 0 | 0 |
| 0 | 0 | 0 |
| 0 | 0 | 0 |
| 0 | 0 | 0 |
| 0 | 0 | 0 |
| 0 | 0 | 0 |
| 0 | 0 | 0 |
