# Supplementary material for: Beyond Ubiquity: Scale-dependent patterns of tardigrade diversity on the Iztaccíhuatl volcano
Source: PLoS One. 2026 Mar 4;21(3):e0343098. doi: 10.1371/journal.pone.0343098 (PMC12959721; doi:10.1371/journal.pone.0343098)
Supplement: S3 Table — (DOCX) [file pone.0343098.s003.docx]

Supporting Information

**Beyond Ubiquity: Scale-dependent patterns of tardigrade diversity on the Iztaccíhuatl volcano,**

Alba Dueñas-Cedillo ^1 #a^, Francisco Armendáriz-Toledano ^2¶*^, Rodolfo Cancino-López ^3^, Jazmín García-Román ^1 #a^, Enrico Alejandro Ruiz ^1¶*^

S3 Table. Abundance matrix of taxa used for alpha diversity analysis and species accumulation curves at different landscape types, (Hill numbers: 0, 1, and 2), performed using the iNext program with a 95% confidence interval and 50 bootstraps.

| Mix F | Meso F | Pinus F | Al grassla | Al tundra |
| --- | --- | --- | --- | --- |
| 92 | 83 | 295 | 122 | 17 |
| 51 | 53 | 133 | 2 | 15 |
| 42 | 32 | 84 | 1 | 6 |
| 14 | 31 | 66 | 1 | 6 |
| 9 | 24 | 59 | 0 | 6 |
| 9 | 12 | 44 | 0 | 3 |
| 5 | 6 | 17 | 0 | 2 |
| 4 | 5 | 10 | 0 | 2 |
| 4 | 2 | 10 | 0 | 1 |
| 3 | 1 | 9 | 0 | 1 |
| 3 | 1 | 8 | 0 | 0 |
| 2 | 1 | 5 | 0 | 0 |
| 1 | 1 | 5 | 0 | 0 |
| 1 | 1 | 3 | 0 | 0 |
| 1 | 1 | 3 | 0 | 0 |
| 1 | 0 | 3 | 0 | 0 |
| 1 | 0 | 3 | 0 | 0 |
| 1 | 0 | 2 | 0 | 0 |
| 1 | 0 | 1 | 0 | 0 |
| 0 | 0 | 0 | 0 | 0 |
| 0 | 0 | 0 | 0 | 0 |
| 0 | 0 | 0 | 0 | 0 |
| 0 | 0 | 0 | 0 | 0 |
| 0 | 0 | 0 | 0 | 0 |
| 0 | 0 | 0 | 0 | 0 |
| 0 | 0 | 0 | 0 | 0 |
| 0 | 0 | 0 | 0 | 0 |
| 0 | 0 | 0 | 0 | 0 |
| 0 | 0 | 0 | 0 | 0 |
| 0 | 0 | 0 | 0 | 0 |
