# Supplementary material for: Beyond Ubiquity: Scale-dependent patterns of tardigrade diversity on the Iztaccíhuatl volcano
Source: PLoS One. 2026 Mar 4;21(3):e0343098. doi: 10.1371/journal.pone.0343098 (PMC12959721; doi:10.1371/journal.pone.0343098)
Supplement: S4 Table — (DOCX) [file pone.0343098.s004.docx]

Supporting Information

**Beyond Ubiquity: Scale-dependent patterns of tardigrade diversity on the Iztaccíhuatl volcano,**

Alba Dueñas-Cedillo ^1 #a^, Francisco Armendáriz-Toledano ^2¶*^, Rodolfo Cancino-López ^3^, Jazmín García-Román ^1 #a^, Enrico Alejandro Ruiz ^1¶*^

S4 Table. Abundance matrix of taxa used for alpha diversity analysis and species accumulation curves in the alpine and nival zones (Hill numbers: 0, 1, and 2), performed using the iNext program with a 95% confidence interval and 50 bootstraps.

| alpine | nival |
| --- | --- |
| 469 | 138 |
| 157 | 17 |
| 104 | 8 |
| 101 | 7 |
| 78 | 6 |
| 73 | 6 |
| 53 | 6 |
| 44 | 6 |
| 32 | 3 |
| 24 | 3 |
| 20 | 2 |
| 20 | 2 |
| 13 | 1 |
| 10 | 1 |
| 7 | 0 |
| 7 | 0 |
| 5 | 0 |
| 4 | 0 |
| 4 | 0 |
| 3 | 0 |
| 3 | 0 |
| 3 | 0 |
| 2 | 0 |
| 2 | 0 |
| 1 | 0 |
| 1 | 0 |
| 1 | 0 |
| 0 | 0 |
| 0 | 0 |
