# Supplementary material for: Beyond Ubiquity: Scale-dependent patterns of tardigrade diversity on the Iztaccíhuatl volcano
Source: PLoS One. 2026 Mar 4;21(3):e0343098. doi: 10.1371/journal.pone.0343098 (PMC12959721; doi:10.1371/journal.pone.0343098)
Supplement: S6 Table — Beta diversity was performed with Total taxonomic beta diversity (total dissimilarity [βsor]) as the sum of its components (turnover [βsim] and nestedness [βnes]). (DOCX) [file pone.0343098.s006.docx]

|  | Sp.1 | Sp.2 | Sp.3 | Sp.4 | Sp.5 | Sp.6 | Sp.7 | Sp.8 | Sp.9 | Sp.10 | Sp.11 | Sp.12 | Sp.13 | Sp.14 | Sp.15 | Sp.16 | Sp.17 | Sp.18 | Sp.19 | Sp.20 | Sp.21 | Sp.22 | Sp.23 | Sp.24 | Sp.25 | Sp.26 | Sp.27 | Sp.28 | Sp.29 | Sp.30 |
| --- | --- | --- | --- | --- | --- | --- | --- | --- | --- | --- | --- | --- | --- | --- | --- | --- | --- | --- | --- | --- | --- | --- | --- | --- | --- | --- | --- | --- | --- | --- |
| Mixed Mixed  forest | 0 | 0 | 1 | 1 | 0 | 0 | 1 | 1 | 1 | 1 | 0 | 1 | 1 | 0 | 0 | 0 | 0 | 0 | 1 | 1 | 1 | 1 | 1 | 1 | 1 | 1 | 1 | 1 | 1 | 0 |
| *Abies religiosa* forest | 1 | 0 | 1 | 1 | 1 | 1 | 0 | 0 | 1 | 0 | 0 | 0 | 0 | 0 | 0 | 0 | 0 | 0 | 0 | 1 | 0 | 1 | 1 | 1 | 0 | 1 | 1 | 1 | 1 | 1 |
| *Pinus hartwegii* forest | 1 | 1 | 1 | 0 | 0 | 0 | 0 | 0 | 1 | 0 | 1 | 0 | 1 | 1 | 0 | 0 | 1 | 1 | 1 | 1 | 1 | 1 | 1 | 1 | 1 | 1 | 1 | 1 | 0 | 1 |
| Alpine grassland | 0 | 0 | 0 | 0 | 0 | 0 | 0 | 0 | 0 | 0 | 0 | 0 | 0 | 0 | 0 | 0 | 0 | 0 | 0 | 0 | 0 | 1 | 1 | 1 | 1 | 0 | 0 | 0 | 0 | 0 |
| Alpine tundra | 0 | 0 | 0 | 0 | 0 | 0 | 0 | 0 | 0 | 0 | 0 | 0 | 0 | 1 | 1 | 1 | 0 | 1 | 1 | 1 | 0 | 1 | 1 | 1 | 0 | 0 | 1 | 0 | 0 | 0 |

Supporting Information

**Beyond Ubiquity: Scale-dependent patterns of tardigrade diversity on the Iztaccíhuatl volcano,**

Alba Dueñas-Cedillo ^1 #a^, Francisco Armendáriz-Toledano ^2¶*^, Rodolfo Cancino-López ^3^, Jazmín García-Román ^1 #a^, Enrico Alejandro Ruiz ^1¶*^

S6 Table. Presence, absence matrix used for beta diversity and two-way cluster analysis by landscape scale, performed using the R program and Past Program. Beta diversity was performed with Total taxonomic beta diversity (total dissimilarity [βsor]) as the sum of its components (turnover [βsim] and nestedness [βnes]).

Sp.1 *Adropion onorei*

Sp.2 *Adropion scoticum*

Sp.3 *Claxtonia* cf*. maucci*

Sp.4 *Doryphoribius* sp.

Sp.5 *Diphascon* cf. *claxtonae*

Sp.6 *Diphascon* cf. *dastychi*

Sp.7 *Diphascon* cf. *faialense*

Sp.8 *Diphascon* cf. *mitrense*

Sp.9 *Diphascon* cf. *pingue*

Sp.10 *Diphascon* cf. *pingueforme*

Sp.11 *Diphascon* cf. *victoriae*

Sp.12 *Hypsibius* 200 (sp nov 1)

Sp.13 *Hypsibius* 200 (sp nov.2)

Sp.14 *Hypsibius* 200 (sp nov.3)

Sp.15 *Hypsibius* 200 (sp nov..4)

Sp.16 *Hypsibius* 200 (sp nov.5)

Sp.17 *Hypsibius* cf. *pedrottii*

Sp.18 *Hypsibius* cf. *microps*

Sp.19 *Hypsibius* cf. *pallidus*

Sp.20 *Macrobiotus hufelandi* OCA *patagonicus*

Sp.21 *Macrobiotus* OCA *lissostomus*

Sp.22 *Mesobiotus* aff. *harmsworthi*

Sp.23 *Milnesium (tardigradum)* sp. [3-3, 3-3]

Sp.24 *Minibiotus sidereus*

Sp.25 *Minibiotus citlalium*

Sp.26 *Pseudechiniscus* (*Pse.*) sp*.*

Sp.27 *Paramacrobiotus* sp

Sp.28 *Degmion nodulosus*.

Sp.29 *Ramazzottius*s
