# Supplementary material for: Beyond Ubiquity: Scale-dependent patterns of tardigrade diversity on the Iztaccíhuatl volcano
Source: PLoS One. 2026 Mar 4;21(3):e0343098. doi: 10.1371/journal.pone.0343098 (PMC12959721; doi:10.1371/journal.pone.0343098)
Supplement: S7 Table — This analysis was performed in Past software. (DOCX) [file pone.0343098.s007.docx]

S7 Table. Presence, absence matrix used for the two-way cluster analysis by elevation zones. This analysis was performed in Past software.

|  | Sp. 1 | Sp. 2 | Sp. 3 | Sp. 4 | Sp. 5 | Sp. 6 | Sp. 7 | Sp. 8 | Sp. 9 | Sp. 10 | Sp.  11 | Sp. 12 | Sp. 13 | Sp. 14 | Sp. 15 | Sp.  16 | Sp. 17 | Sp. 18 | Sp. 19 | Sp. 20 | Sp. 21 | Sp. 22 | Sp. 23 | Sp. 24 | Sp. 25 | Sp. 26 | Sp. 28 | Sp. 29 |
| --- | --- | --- | --- | --- | --- | --- | --- | --- | --- | --- | --- | --- | --- | --- | --- | --- | --- | --- | --- | --- | --- | --- | --- | --- | --- | --- | --- | --- |
| alpine | 1 | 1 | 1 | 1 | 1 | 1 | 1 | 1 | 1 | 1 | 1 | 1 | 0 | 0 | 1 | 1 | 1 | 1 | 1 | 1 | 1 | 1 | 1 | 1 | 1 | 1 | 1 | 1 |
| nival | 1 | 1 | 0 | 0 | 0 | 0 | 0 | 0 | 0 | 0 | 0 | 1 | 1 | 1 | 0 | 1 | 1 | 1 | 0 | 1 | 1 | 1 | 1 | 0 | 1 | 1 | 0 | 0 |

Sp.1 *Adropion onorei*

Sp.2 *Adropion scoticum*

Sp.3 *Claxtonia* cf*. maucci*

Sp.4 *Doryphoribius* sp.

Sp.5 *Diphascon* cf. *claxtonae*

Sp.6 *Diphascon* cf. *dastychi*

Sp.7 *Diphascon* cf. *faialense*

Sp.8 *Diphascon* cf. *mitrense*

Sp.9 *Diphascon* cf. *pingue*

Sp.10 *Diphascon* cf. *pingueforme*

Sp.11 *Diphascon* cf. *victoriae*

Sp.12 *Hypsibius* 200 (sp nov 1)

Sp.13 *Hypsibius* 200 (sp nov.2)

Sp.14 *Hypsibius* 200 (sp nov.3)

Sp.15 *Hypsibius* 200 (sp nov..4)

Sp.16 *Hypsibius* 200 (sp nov.5)

Sp.17 *Hypsibius* cf. *pedrottii*

Sp.18 *Hypsibius* cf. *microps*

Sp.19 *Hypsibius* cf. *pallidus*

Sp.20 *Macrobiotus hufelandi* OCA *patagonicus*

Sp.21 *Macrobiotus* OCA *lissostomus*

Sp.22 *Mesobiotus* aff. *harmsworthi*

Sp.23 *Milnesium (tardigradum)* sp. [3-3, 3-3]

Sp.24 *Minibiotus sidereus*

Sp.25 *Minibiotus citlalium*

Sp.26 *Pseudechiniscus* (*Pse.*) sp*.*

Sp.27 *Paramacrobiotus* sp

Sp.28 *Degmion nodulosus*.

Sp.29 *Ramazzottius*
