# Supplementary material for: Beyond Ubiquity: Scale-dependent patterns of tardigrade diversity on the Iztaccíhuatl volcano
Source: PLoS One. 2026 Mar 4;21(3):e0343098. doi: 10.1371/journal.pone.0343098 (PMC12959721; doi:10.1371/journal.pone.0343098)
Supplement: S8 Table — (DOCX) [file pone.0343098.s008.docx]

Supporting Information

**Beyond Ubiquity: Scale-dependent patterns of tardigrade diversity on the Iztaccíhuatl volcano,**

Alba Dueñas-Cedillo ^1 #a^, Francisco Armendáriz-Toledano ^2¶*^, Rodolfo Cancino-López ^3^, Jazmín García-Román ^1 #a^, Enrico Alejandro Ruiz ^1¶^

S8 Table. Presence-absence matrix used for Principal Coordinate Analyses (PCoA) analyses, using Jaccard index in Past software.

|  | Mix F | Meso F | Pinus F | Al grassla | Al tundra | bark | soil | rock | alpine z | nival z |
| --- | --- | --- | --- | --- | --- | --- | --- | --- | --- | --- |
| *Adropion onorei* | 0 | 1 | 1 | 0 | 0 | 1 | 1 | 1 | 1 | 1 |
| *Milnesium (tardigradum)* sp. [3-3, 3-3] | 1 | 0 | 1 | 1 | 0 | 1 | 1 | 1 | 1 | 1 |
| *A scoticum* | 0 | 0 | 1 | 0 | 0 | 0 | 1 | 1 | 1 | 1 |
| *Diphascon* cf. *claxtonae* | 0 | 1 | 0 | 0 | 0 | 1 | 0 | 0 | 1 | 0 |
| *Diphascon* cf. *dastychi* | 0 | 1 | 0 | 0 | 0 | 1 | 0 | 0 | 1 | 0 |
| *Diphascon* cf. *faialense* | 1 | 0 | 0 | 0 | 0 | 1 | 0 | 0 | 1 | 0 |
| *Diphascon* cf. *mitrense* | 1 | 0 | 0 | 0 | 0 | 1 | 0 | 0 | 1 | 0 |
| *Diphascon* cf. *pingue* | 1 | 1 | 1 | 0 | 0 | 1 | 1 | 0 | 1 | 1 |
| *Diphascon* cf. *pingueforme* | 1 | 0 | 0 | 0 | 0 | 1 | 0 | 0 | 1 | 0 |
| *Diphascon* cf. *victoriae* | 0 | 0 | 1 | 0 | 0 | 1 | 0 | 0 | 0 | 1 |
| *Hypsibius* 210 (sp nov. 1) | 0 | 0 | 1 | 0 | 1 | 0 | 1 | 1 | 0 | 1 |
| *Hypsibius* 210 (sp nov. 2) | 0 | 0 | 0 | 0 | 1 | 0 | 1 | 0 | 0 | 1 |
| *Hypsibius* 210 (sp nov. 3) | 0 | 0 | 0 | 0 | 1 | 0 | 1 | 0 | 0 | 1 |
| *Hypsibius* 210 (sp nov. 4) | 0 | 0 | 1 | 0 | 0 | 0 | 1 | 0 | 1 | 1 |
| *Hypsibius* 210 (sp nov. 5) | 0 | 0 | 1 | 0 | 1 | 0 | 1 | 1 | 1 | 1 |
| *Hypsibius* cf. *pedrottii* | 1 | 0 | 1 | 0 | 1 | 1 | 1 | 1 | 1 | 1 |
| *Hypsibius* cf. *microps* | 1 | 1 | 1 | 0 | 1 | 1 | 1 | 1 | 1 | 1 |
| *Hypsibius* cf. *pallidus* | 1 | 0 | 1 | 0 | 0 | 1 | 1 | 0 | 1 | 1 |
| *Degmion nodulosus* | 0 | 1 | 1 | 0 | 0 | 1 | 1 | 0 | 1 | 1 |
| *Macrobiotus hufelandi* OCA *patagonicus* | 1 | 1 | 1 | 1 | 1 | 1 | 1 | 1 | 1 | 1 |
| *Macrobiotus hufelandi* OCA *lissostomus* | 1 | 1 | 1 | 1 | 1 | 1 | 1 | 1 | 1 | 1 |
| *Mesobiotus* aff. *harmsworthi* | 1 | 1 | 1 | 1 | 1 | 1 | 1 | 1 | 1 | 1 |
| *Minibiotus sidereus* | 1 | 1 | 1 | 0 | 0 | 1 | 1 | 0 | 1 | 1 |
| *Minibiotus citlalium* | 1 | 1 | 1 | 0 | 1 | 1 | 1 | 1 | 1 | 1 |
| *Paramacrobiotus* sp. | 1 | 1 | 0 | 0 | 0 | 1 | 1 | 0 | 1 | 0 |
| *Pseudechiniscus* (*Pse.*) sp*.* | 1 | 1 | 1 | 0 | 0 | 1 | 0 | 1 | 1 | 1 |
| *Claxtonia* cf. *maucci* | 1 | 1 | 1 | 0 | 0 | 1 | 1 | 0 | 1 | 1 |
| *Doryphoribius* sp*.* | 1 | 1 | 0 | 0 | 0 | 1 | 0 | 0 | 1 | 0 |
